# A Descriptive Analysis of Febrile Seizure Hospitalizations in Children with Congenital Heart Disease in the United States

**DOI:** 10.7759/cureus.44128

**Published:** 2023-08-25

**Authors:** Ebenezer O Adebiyi, Ruth Y Eletta, William Ogedengbe, Oreoluwa J Kolade-Ernest, Juanita Hunter

**Affiliations:** 1 Pediatric Cardiology, University of Texas Health Science Center, Houston McGovern Medical School, Houston, USA; 2 Pediatrics, Woodhull Medical Center, New Jersey, USA; 3 Medicine and Surgery, Lagos State University Teaching Hospital (LUTH), Lagos, NGA; 4 Pediatrics, State University of New York (SUNY) Downstate Health Sciences University, New York, USA; 5 Pediatric Cardiology, University of Miami Miller School of Medicine, Jackson Memorial Hospital, Miami, USA

**Keywords:** united states of america, united states, pediatrics, congenital heart disease (chd), hospitalized patients, febrile seizure

## Abstract

Background: Febrile seizure (FS) is the most common convulsive disorder in children. This study analyzed the national proportion of congenital heart disease (CHD) and hospital resource utilization among children admitted for FSs in the U.S.

Methods: This is a retrospective cross-sectional analysis of pediatric patients up to six years with a primary diagnosis of FS in 2016 and 2019 using the Kids Inpatient Database (KID). The demographic, hospital, and clinical characteristics of children with and without CHD were compared using the chi-square test for categorical variables and linear regressions for continuous variables. Multivariate logistic analysis was conducted to evaluate the impact of CHD on the mean length of hospital stay.

Results: An estimated 10,039 children were admitted with the primary diagnosis of FS. Out of these, 117 (1.2%) had a discharge diagnosis of CHD. The mean age for children with and without CHD was 1.4 years (SD 1.60) and 1.5 years (SD 1.501), respectively. Children with CHD who required hospitalization for FS had longer mean lengths of hospital stay (2.1 days vs. 1.6 days), with an adjusted odd ratio of 0.43 (95% CI: 0.07-0.99; p-value: 0.017). Similarly, the hospital charges for children with CHD were higher than those without CHD ($30,960.28 vs. $21,005.11).

Conclusion: Children with CHD who required inpatient admission for FSs in the U.S. were associated with increased length of hospital stay and higher resource utilization when compared with those without CHD. This highlights the need for preventive measures among this vulnerable population.

## Introduction

Febrile seizures (FSs) are the most common convulsive disorder in children, occurring in 2-5% of those in early childhood [1]. Viral infections remain the most common cause of FS in children. In addition, many other factors, such as genetic components, family history, and underlying comorbid diseases, are significantly associated with the risk of FS. In this study, we evaluated the influence of congenital heart disease (CHD) on FS-related hospitalization, as CHD is one of the crucial comorbid risk factors for seizures in children.

CHD represents one of the most common congenital disabilities in children and occurs in approximately 1% of all births [2]. Children with critical CHD are particularly vulnerable to the development of seizures [3]. The brain undergoes a period of optimal growth during infancy and any insult to the developing brain during this period is associated with an increased risk of adverse neurologic outcomes [4]. The injury may result from the severe hypoxia and hypoperfusion often associated with their primary cardiac pathologies or from complications during surgical intervention, such as cardiopulmonary bypass surgeries.

Many studies have detailed the impact of critical CHD on developing seizure disorders in children. A population-based cohort study found the cumulative incidence of epilepsy to be about 5% in patients with CHD, both before and after surgical intervention, by the age of 15 years [3]. Another prospective study reported the incidence of epilepsy to be about 3.1% among children with CHD and increased incidence was associated with surgical complexity, prolonged hospital stay, and extracorporeal membrane oxygenation [5]. Still, none of these studies has examined the prevalence of CHD among children admitted for FSs, which usually do not require hospitalization. This study aims to evaluate the national proportion of CHD among children primarily hospitalized for FS in the U.S. and analyze hospital resource utilization among these children.

The findings of this study will provide further insights into both demographic and clinical characteristics of children with or without CHD who were admitted for FS and assess how their disease condition impacts their hospital outcomes.

## Materials and methods

Study design and data source

This is a cross-sectional study of pediatric patients with a primary diagnosis of FS in 2016 and 2019 using the Kids Inpatient Database (KID). The KID is a Healthcare Cost and Utilization Project (HCUP) database operated by the Agency for Healthcare Research and Quality [6]. It is the largest publicly available database on pediatric hospital admissions in the U.S. The database samples 80% of all pediatric hospital discharges to produce extensive data, thus increasing statistical power for analyzing rare medical diseases among hospitalized children in the U.S. It includes all-payer admissions from children’s hospitals in 48 U.S. states and the District of Columbia. All the KID data contain de-identified patient information. Both 2016 and 2019 included more than 6.1 million unweighted discharges, which were weighted to 12 million pediatric hospital admissions in the U.S. The weighted discharge, based on the complex sample design of the HCUP data, allows for generating nationally representative data. 

Study sample

This study included children younger than seven years of age with the primary diagnosis of FS based on the International Classification of Diseases, tenth edition, Clinical Modification (ICD-10 CM) (Table 3 of Appendix). The ICD-10 codes included are R56.0, R56.00, and R56.01. Previous studies using the HCUP data applied the ICD-9 version of these codes to identify FS [7,8]. To limit the diagnoses to those without any concurrent neurological or neuromuscular disease, we excluded those with secondary diagnoses relating to nervous system diseases designated as G00-G99 in the ICD-10 CM codes. These include inflammatory diseases of the central nervous system (CNS) (G00-G09), systemic atrophies primarily affecting the CNS (G10-G14), extrapyramidal and movement disorders (G20-G26), degenerative nervous system disease (G30-G32), demyelinating CNS disease (G35-G37), episodic and paroxysmal disorders (G40-G47), nerve, nerve root, and plexus disorders (G50-G59), polyneuropathies and other conditions of the peripheral nervous system (G60-G65), myoneural junction and muscle disease (G70-G73), cerebral palsy, other paralytic syndromes (G80-G83), and other disorders of the nervous system (G89-G99).

Children with CHD were identified using the ICD-10 codes (as shown in the appendix). Similar to an approach used in a previous study, we excluded diagnoses related to patent ductus arteriosus, to minimize its effects, which are usually insignificant after infancy [9]. We adopted the American Heart Association’s current Adult CHD anatomy classification guideline to group CHD into simple, moderate, and complex [10]. A similar approach was used in a previous scientific publication [11].

Descriptive variables

The KID data provided the essential demographic variables included in this study. The mean age of the children was included, and gender was classified as male and female based on the HCUP classification. The database provides six racial groups (white, black, Hispanic, Asian/Pacific Islander, Native American, and others), which we reclassified as white, black, Hispanic, and others because of the relatively small number of those in the Asian/Pacific Islander and Native American groups. The socioeconomic status included the primary payment source (insurance) and median household income national quartile for the patient's zip code. We reclassified primary insurance as private insurance or insured through Medicaid. Other patient characteristics in the database were the mean length of hospital stay and total hospital charges. The HCUP database contained information about each hospital's characteristics. The hospital characteristics included in this study are region, bed size, and teaching status. 

Statistical analysis

Descriptive characteristics were generated for the demographic, hospital, and clinical characteristics and expressed as percentages or means. Children with and without CHD were compared using the chi-square test for categorical variables and linear regressions for continuous variables. The Consumer Price Index Inflation calculator maintained by the U.S. Bureau of Labor Statistics was employed to calculate the adjusted mean total hospital charges to account for inflation over the studied years [12]. The mean differences in the length of hospital stay and total hospital charges for pediatric FS admissions were compared between those with and without CHD. Multivariate logistic analysis was conducted to evaluate the impact of CHD on the mean length of stays among children admitted for FS using the patients' demographic, hospital, and clinical characteristics as confounding predictors. Stata software version 17.0 (StataCorp LLC, College Station, TX, USA) was used for all statistical analyses of this study. The p-values were two-sided, and 0.05 was set as the cut-off for statistical significance. All analyses in this study employed the complex sampling design provided by the HCUP in terms of stratification, clustering, weighing, and selection of the study sample.

## Results

Overall, there were 10,039 pediatric hospital admissions for FS in 2016 and 2019 in the U.S.; 117 (1.2%) of these patients had CHD, while 9922 (98.8%) did not. Table 1 describes the demographic and clinical characteristics of pediatric hospital admissions for FS in this study. The same percentages of children with or without CHD were admitted for simple FS. Complex FS was slightly more common in children with than without CHD (62.9% versus 62.8%).

**Table 1:** Demographic and clinical characteristics of pediatric hospital admissions for FS in 2016 and 2019 *Adjusted based on the U.S Bureau of Labor Statistics Consumer Price Index (July 2016-October 2022); ۸HCUP does not permit reporting a cell size less than or equal to 10 SD, standard deviation; FS, febrile seizure

	Congenital heart disease				
	Yes		No		
	N	%	n	%	p-value
Total number of FS admission	117	1.2	9922	98.8	
FS types					0.987
Simple	43	37.1	3688	37.1	
Complex	74	62.9	6234	62.8	
Mean age in years (SD)	1.4(1.6)	-	1.5(1.501)	-	0.309
Weekend admission					0.867
No	86.12	73.36	7194	72.51	
Yes	31.27	26.64	2728	27.49	
Sex					0.313
Female	59	50.12	4405	44.4	
Male	58	49.88	5517	55.6	
Race					0.549
White	37	33.7	3787	40.8	
Black	20	18.6	1729	18.6	
Hispanic	30	27.6	2287	24.6	
Other races	22	20.0	1475	15.9	
Insurance types					0.034
Medicaid	84	72.3	5914	59.7	
Private	23	19.8	3331	33.6	
Others	<11^۸^	NA	666	6.7	
Zip code income quartile (percentile)					0.257
0-25	47.77	42.78	3272	33.38	
26-50	20.36	18.24	2405	24.53	
51-75	25.73	23.04	2213	22.58	
76-100	17.79	15.94	1914	19.52	
Hospital region					0.324
Northeast	16.2	13.80	2125	21.42	
Midwest	29.53	25.16	1998	20.13	
South	40.03	34.10	3338	33.64	
West	31.63	26.94	2462	24.81	
Hospital bed size					0.518
Small	16.58	14.12	1100	11.09	
Medium	18.23	15.53	1950	19.65	
Large	82.58	70.35	6872	69.26	
Hospital teaching status					0.355
Non-teaching	9.758	8.31	1142	11.51	
Teaching	10 7.6	91.69	8780	88.49	
Mean length of stay (days)	2.1	-	1.6	-	0.009
Mean total hospital charges ($)*	30,960.28		21,005.11		0.004
Year					0.455
2016	66	56.6	5213	52.5	
2019	51	43.4	4709	47.5	

The mean age for children with and without CHD was 1.4 years (SD 1.60) and 1.5 years (SD 1.501), respectively. A higher percentage of children with CHD were female (50.1%) compared with a higher percentage of males in those without CHD (55.6%). Most pediatric hospital admissions for FSs were Whites, accounting for 33.7% of patients with CHD (37) and 40.8% of patients without CHD (3787).

Approximately 19% of the cohort with CHD were of black race and 27.6% were Hispanic. Other races accounted for the remaining 22 patients (20%) with CHD. A similar distribution was noted in the patients without CHD. Children of the black race without CHD were 18.6% of the sample (1729) and Hispanics were 24.6%, (2287); the remaining 15.9% (1475 patients) were of other races. 

Patients with CHD and Medicaid insurance were 72.3% of the sample (84); those with the private insurance type were 19.8% (23). Among patients without CHD, 59.7% (5914) were on Medicaid, 33.6% (3331) were on private insurance, and the rest were on other types of payment. Though not significant in the bivariate analysis, there was a high correlation between the income quartile of patients and hospital admission for FS in children both with and without CHD. Children with CHD with income quartile within the range of 0-25 made up 42.78% of the admitted sample (48); those within the range of 26-50 were 18.24% (20); those within the range of 51-75 were 23.04% (26); and those within the range of 76-100 were 15.94% (18). For children without CHD, 33.38% (3272) were in the 0-25 income quartile range, 24.53% (2405) were in the 26-50 range, 22.58% (2213) were in the 51-75 range, and 19.52% (1914) were in the 76-100 range. 

Among patients with CHD, 26.64% (31) were hospitalized during the weekends, while the rest (73.36%) were hospitalized during the weekday. Among those without CHD, 72.51% (7194) were hospitalized during the weekdays, while 27.49% (2728) were hospitalized on weekends. Most of the patients with CHD and FS were admitted to teaching hospitals (107; 91.69%), while non-teaching hospitals managed the rest (8.31%). Of patients without CHD, 88.49% (8780) were admitted to teaching hospitals, while 11.51% (1142) were managed by non-teaching hospitals.

Febrile seizure and length of hospital stay

**Figure 1:**
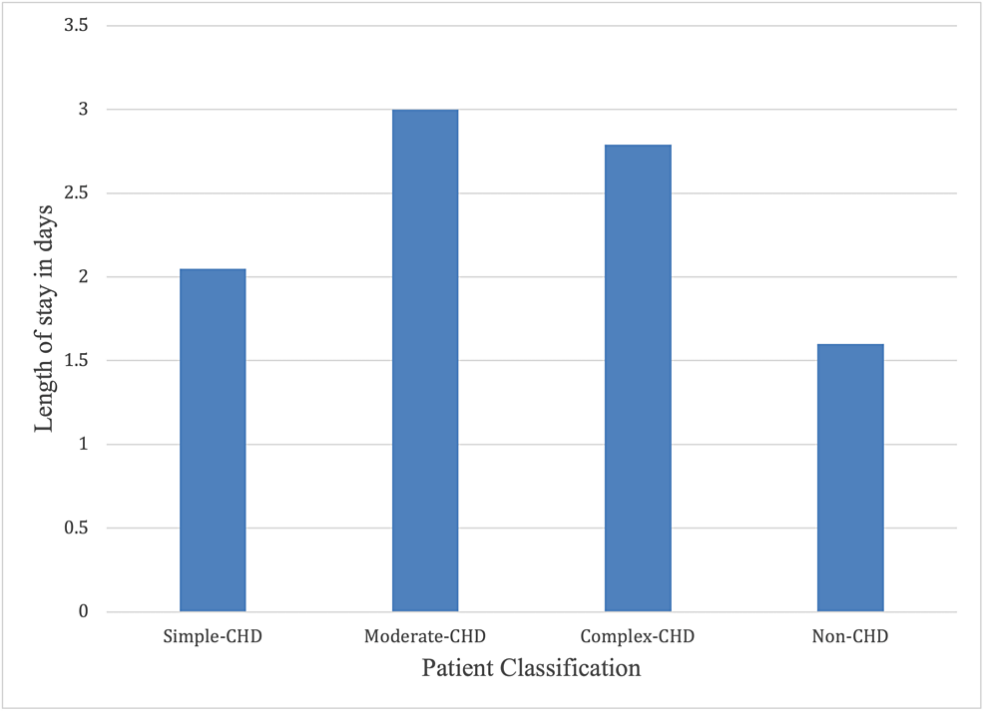
Length of hospital stay for children with FS among different categories of CHD FS, febrile seizure; CHD, congenital heart disease

At 2.1 days, patients with CHD and FSs had an increased mean length of hospital stay (LOS) than those without CHD (1.6 days). In the multivariable logistic regression models (Table 2), children with CHD and FS had an increased risk for longer LOS than those without CHD (aOR 0.43, CI 0.077-0.792, p-value: 0.017). Further analysis of the mean LOS (days) among the three classes of CHD, as represented on the chart (Figure 1), revealed that children with simple CHD had the shortest mean LOS, with 2.05 days, those with moderate CHD had the longest mean LOS at three days, while those with complex CHD had a mean LOS of 2.79 days. 

**Table 2:** Multivariate logistic predictors of length of stay following hospitalization for FS in 2016 and 2019 Weekday admission, male sex, whites, Medicaid insurance type, northeast hospital region, small-sized and non-teaching hospitals were set as reference variables. aOR, adjusted odds ratio; FS, febrile seizure; CI, confidence interval

Variables	aOR	CI	p-value
Congenital heart disease	0.43	0.077-0.792	0.017
Age	-0.05	(-0.075)-(-0.312)	<0.001
Weekend admission	0.02	0.03-0.08	0.430
Sex			
Female	0.03	-0.02-0.09	0.241
Race			
Black	0.05	-0.03-0.14	0.208
Hispanic	0.03	-0.04-0.10	0.451
Other races	0.02	-0.06-0.10	0.618
Insurance types			
Private	-0.12	(-0.18-(-0.62)	0.001
Others	-0.20	(-0.32)-(-0.80)	0.001
Hospital region			
Midwest	-0.34	-0.14-0.07	0.162
South	0.08	-0.02-0.19	0.141
West	-0.21	(-0.32)-(-0.09)	0.001
Hospital bed size			
Medium	0.11	-0.14-0.27	0.162
Large	-0.01	-0.15-0.19	0.829
Hospital teaching status			
Teaching	-0.04	-0.14-0.06	0.423

Febrile seizure and total hospital charges

**Figure 2:**
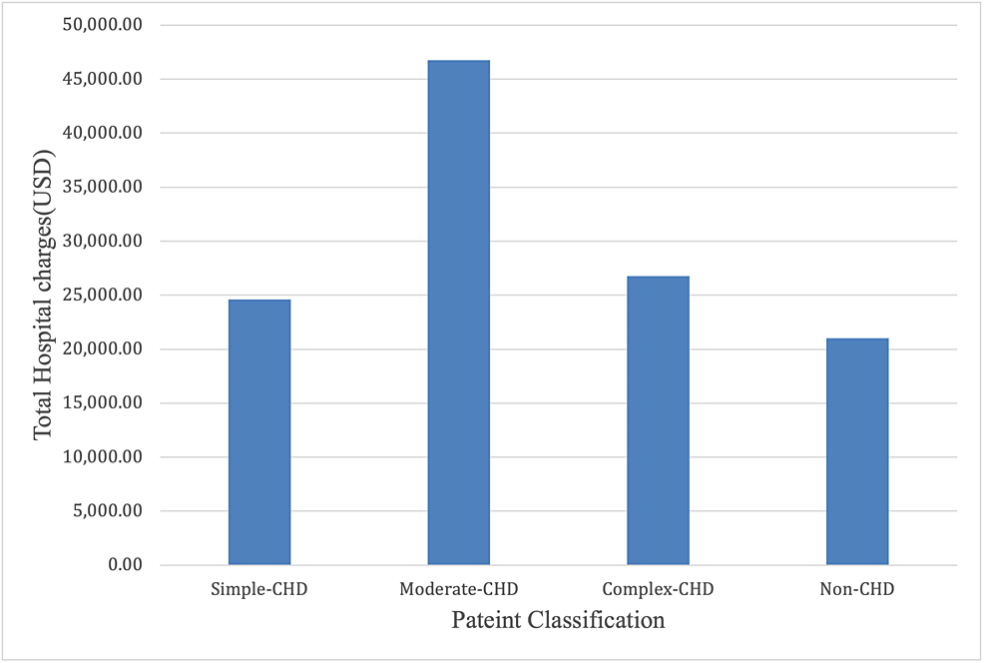
Inflation-adjusted total hospital charges among different categories of CHD CHD, congenital heart disease

Figure 2 shows the inflation-adjusted total charge for pediatric hospital admissions for FS in 2016 and 2019. The mean inflation-adjusted total hospital charges were significantly higher in patients with CHD ($40,518.77) than for those without CHD ($21,005.11). The inflation-adjusted total charge for children with simple CHD was $30,468.48. In contrast, those with moderate CHD and complex CHD were charged $57,913.49 and $33,174.34, respectively.

## Discussion

CHD is prevalent in about 1.3% of children in the U.S. [13]. Children with CHD and other chronic medical conditions are at increased risk of FSs, which can result from complications of viral infections to which they are predisposed [9,14]. Fever associated with viral infections increases seizure risk by increasing neuronal excitability and lowering the seizure threshold [15]. Most children with FS will usually not require hospitalization or, when hospitalization is required, the hospital stay will not be prolonged. Our study found an estimated 1.2% of all FS hospitalizations in the U.S. occurred in children with CHD in 2016 and 2019. Our study also found that patients with CHD admitted for FS had an increased length of hospital stay and total hospital charges than children without CHD. 

Our analysis found a similar distribution in terms of the proportion of CHD among children hospitalized for FS and that of the prevalence of CHD among the general population of children in the U.S. While we found 1.2% of children with CHD among those with FS-related hospitalization, Gilboa et al. (2016) found 1.3% of U.S. children living with CHD in 2010 [13]. Therefore, FS hospitalization may not occur more frequently in children with CHD than those without it given the similar proportion. When children with CHD present with a seizure, it is crucial to determine whether there is a background seizure disorder or other underlying neurological impairment before the diagnosis of FS can be entertained. Though there are no studies, to our knowledge, on the influence of CHD on FS hospitalization, studies have shown that children with CHD are at increased risk of developing seizure disorders and other neurologic complications. Leisner et al. described an increased risk of epilepsy in children with CHD who did not undergo surgery [4]. Claessens et al. (2017) described altered antenatal cerebral circulation and acquired brain injury in neonates with CHD [3]. Also, patients with CHD are at increased risk of acquired strokes and poor neurocognitive functions [3].

We found out that children with CHD who required hospitalization for FS were younger than those without CHD, though this relationship was not significant in the bivariate analysis. Okubo et al. (2017) studied FSs in hospitalized children using the older version of the KID data we used for our analysis [7]. They found the mean age of children hospitalized for FS to be 1.4 years in 2012. This is similar to 1.4 years and 1.5 years for children with and without CHD, respectively, in our study. As stated above, children with congenital heart may have structural defects that cause insufficient oxygen delivery to the brain which then lowers their seizure threshold and predispose them to FS at an earlier age. The clustering of FS among certain ethnic/racial and geographic distributions has raised the possibility of genetic predisposition to FS. A previous study by Okubo et al. (2017) showed that black and Hispanic children were overrepresented among hospitalized children with FS [7]. Similarly, a disproportionate number of black and Hispanic children were admitted for FS among those with or without CHD in our study.

The hospital stay was longer in children with FS and CHD in this study, with a mean stay of 2.1 days, compared to a mean stay of 1.6 days in patients with FS but without CHD. This is most likely due to the complexity of the disease, various comorbidities, the possible need for surgical intervention, and the difference in approach to the management of seizures in children with CHD. In keeping with this, children with FS and moderate or severe forms of CHD had increased lengths of hospital stay compared to children with FS and simple forms of CHD. A study by Mehnaz et al. (2006 ) showed that the duration of hospitalization was longer in children with CHD compared to other predisposing factors [16]. 

Children with FS and CHD had higher hospital charges ($30,960.28) than those without CHD ($21,005.22), directly proportional to their longer LOS. Other contributing factors may include the need for diagnostic or interventional procedures and the complexity of the management of CHD. 

To our knowledge, this is the first study to analyze FS hospitalization among children with CHD. We have used nationally representative samples of more than 12 million pediatric hospital admissions to evaluate the prevalence of CHD among children admitted for FS and resource utilization among them. The large sample size in this study should allow for appropriate generalization of the findings of this study. 

Our findings must be interpreted in the context of some limitations. There was no information on the medications used before, during, or after admission. The HCUP data does not contain pertinent health records, such as detailed history, physical examination, and other important medical investigations performed during hospitalization. The HCUP data was obtained using ICD-10 CM codes for billing. Hence, there is a possibility of human error in the classification of the disease conditions. Furthermore, the database has information only on the visit level, and it is not possible to track patients' outcomes over time. This research pertains to patients admitted to a hospital, and its findings may not be broadly applicable to children who did not require hospitalization.

## Conclusions

Children with CHD who required hospitalization for FSs had increased length of hospital stay and higher resource utilization when compared with those without CHD. This supports the need for preventive measures among this vulnerable population and further study into other factors that may play a role in managing FSs in patients with CHD. Further prospective studies on non-hospitalized children will provide insights into the incidence of FS among children with CHD. 

## Appendices

**Table 3:** ICD codes used in the identification and classification of CHD ICD-10 CM, International Classification of Diseases, tenth edition, Clinical Modification; CHD, congenital heart disease

	ICD-10 CM codes
CHD	Q20.xx to Q26.xx Excluded: PDA: Q25.0
CHD classifications	
Simple CHD	
Isolated ventricular septal defect	Q21.0
Isolated atrial septal defect	Q21.1
Isolated congenital valve disease	Q22.0, Q22.1, Q22.2, Q22.3, Q22.4, Q22.8, Q22.9, Q23.0, Q23.1, Q23.2, Q23.3, Q25.8
Moderate CHD	
Tetralogy of fallot	Q21.3, Q21.8
Ebstein’s anomaly	Q22.5
Aortic anomalies	Q25.1, Q25.21, Q25.219
Atrioventricular septal defect	Q21.2
Partial anomalous pulmonary venous connection	Q26.3, Q26.4
Complex CHD	
Complex arterial and venous connections	Q20.0, Q26.2
Single ventricles	Q20.1, Q20.2, Q20.4, Q22.6, Q23.4
Transposition of great arteries	Q20.3, Q20.5
